# Twenty years of community-based disaster risk reduction experience from a dryland village in Indonesia

**DOI:** 10.4102/jamba.v10i1.502

**Published:** 2018-05-03

**Authors:** Jonatan A. Lassa, Yos Boli, Yulius Nakmofa, Silvia Fanggidae, Alex Ofong, Herman Leonis

**Affiliations:** 1 Emergency and Disaster Management Studies, Charles Darwin University, Australia; 2 Bank of Indonesia, Kupang, Indonesia; 3 Disaster Management Community Association, Perhimpunan Masyarakat Penanganan Bencana, Indonesia; 4 Yayasan PIKUL, Kupang, Indonesia; 5 National Task Team Provincial Legislative Office, Kupang, Indonesia

## Abstract

Academics and practitioners often argue indirectly that all the roads to community resilience should be paved with community-based disaster risk reduction (CBDRR) approach. Community-based approach to resilience building has been a discursive material that appeals many disaster management players including international donors, non-governmental organisations and high-level government officials as well as politicians. Some researchers argue that CBDRR is the foundation of disaster risk governance. Unfortunately, globally, there is lack of studies on long-term and real-world experience of CBDRR. This article addresses this research gap by providing insights of CBDRR activities from a village in eastern Indonesia based on long-term studies. The adoption of CBDRR approach in Indonesia took place in the late 1990s and the authors have been part of the early adopters of the framework. Using longitudinal participant observations, this research combined qualitative and quantitative data collected during 1998–2017. It shows the rise and fall of a community responding to disaster risks over time. The article further highlights stories of frustrations and celebrations that surround CBDRR activities implemented by one local community in a dryland village in eastern Indonesia.

## Introduction

Community-based approach (CBA) has been a discursive material that appeals to most international donors, non-governmental organisations and high-level government officials (Blaikie 2006). In disaster studies, there is an unwritten consensus that all the ways to achieve resilience, communities must now be paved with community-based disaster risk reduction (CBDRR) framework. Some scholars have argued that CBDRR has been the foundation of disaster risk management (DRM) at societal scale (Zhang, Yi & Zhao 2012).

Community-based approaches to reduce disaster risk have been known by various different names. Some called it community-based disaster risk management (CBDRM), community-based disaster management (CBDM), community-driven disaster risk reduction (CBDRR), community-based disaster preparedness (CBDP), community-driven disaster risk management (CDDRR), community-managed disaster risk reduction (CMDRR) and community-managed disaster risk management (CMDRM). Some have been hazard specific such as community-based flood risk management (CBFRM) or CBDP and a dozen more combinations of wordings. These abbreviations are basically pointing to the earlier concepts of participatory learning and action for DRM (see Von Kotze & Holloway 1996).

These terms are not entirely jargons. For example, the idea of CMDRR puts emphasis largely on communities’ control of disaster risk reduction processes (Dube 2015). By ‘community-managed’, some non-governmental organisations (NGOs) have argued that CMDRR provides more explicit messages of community-controlled disaster risk reduction (DRR) where communities are the managers while external parties such as NGOs are the facilitators (Cordaid & IIRR 2011; IIRR & Cordaid 2013).

Community-based disaster risk reduction concepts have been appealing to both academic and professional communities. Earlier publication of CBDRR came mainly from scholar-practitioners. For example, both Maskrey (1984, 1989) and Von Kotze and Holloway (1996) were the most notable explicit works on CBA to DRM, and both works have been closely associated with Oxfam. Non-governmental organisations such as Oxfam have been the trend setter in the earlier formation of CBDRR frameworks. Some have claimed that history of CBDRR can be traced back to 1980s where the La Red (the Network for Social Studies on Disaster Prevention in Latin America) coined the term *CBDMit* (*community-based disaster mitigation*) (Maskrey 1984, 1989, 2011). In Asian context, the term emerged from two parallel processes in two different settings: Firstly, in South Asia where Duryog Nivaran, a networked organisation, started an alternative version of inclusive DRM and secondly, with the birth of Asian disaster preparedness (ADPC) in 1995, supported by the Office of US Foreign Disaster Assistance (OFDA) (Lorna 2002).

Relying on solid academic works from the United States, community-based approaches received greater attention after World War II despite the dominant focus remaining on reactive response (Phiri 2015). Scientific work on CBA in other disciplines can be traced back to the late 1960s and the 1970s. Community psychologists and family researchers have worked on questions such as how CBA could solve families under stress (Burns & Friedman 1976). Education studies have been reflecting on the utilisation of CBA to promote global education (Woyach & Remy 1982) and law-related education (Naylor 1977). Community-based approaches, later were promoted in the public health sector and demographic studies (e.g. Amolo 1979; Krannich & Krannich 1980; Ruddock 1980). Its uses in public health communities can be traced a decade earlier (see Palmiere 1968).

Learning from the use of CBA in other fields (e.g. natural resource management and development studies – see Blaikie 2006), the proponents perceived CBDRR concepts as a very appealing concept for a number of reasons. Firstly, its proximity to at-risk and vulnerable communities can be privileged by CBDRR. Disaster risk distributions tend to be skewed towards the marginal and most vulnerable groups (Blaikie et al. 1994). Therefore, working with vulnerable people can pre-empt the biases of top-down top-risk identification and prioritisation. Secondly, both scholars and academics have recently shown that locally-managed risk can protect the lives and assets of vulnerable communities (Blaikie et al. 1994). Thirdly, CBDRR attracts inclusion of actors and different types of knowledge. Vulnerable communities and marginalised indigenous communities have a role to play in CBDRR. Grassroots communities are equally seen as the guardians of societal risks as they are working hand in hand with NGOs, governments and the private sectors (Van Niekerk et al. 2018).

**FIGURE 1:**
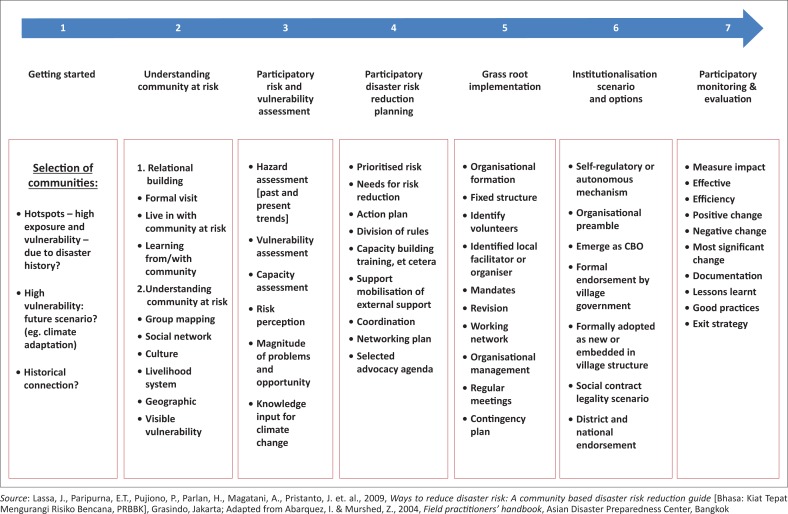
Processes and tools of community-based disaster risk reduction frameworks.

The adoption of a CBDRR approach in Indonesia took place in the late 1990s. There is still a lack of studies on long-term application of CBDRR at the grassroots levels. Practitioners, especially those who come from international and national organisations, often have been constrained by their project and resource settings that prevent them from having long-term engagements with local communities. The process of getting in getting out (GIGO) or flying in flying out (FIFO) by non-governmental organisations has been a regular phenomenon as their mission has been always temporary and short-term because of limited resources. Many manuals and models largely framed CBDRR as a series of events that start from the point where an NGO comes from outside to help a community at risk. Community selection is made based on historical characteristics of disaster risk. The next steps include establishing a basic understanding of the selected communities, building trust (see Figure 1), conducting detailed participatory disaster risk assessment processes that inform community DRM planning, followed by capacity building and CBDRR and/or community-led implementation, as well as ensuring communities to conduct monitoring and evaluation (e.g. Abarquez & Murshed 2004). The process will end with an exit strategy (Lassa et al. 2009).

This article mainly aims at telling stories about CBDRR practices informed by documented knowledge and experiences from an NGO that has been working with the communities and local governments in the Toineke village in eastern Indonesia. We asked: How can a community-based risk reduction project contribute to long-term social change? Sub-questions include the following: How risk and vulnerabilities evolve in a village over a long period of time?; How risks and social problems are perceived, identified, ranked and prioritised over time?; What actions did the communities take over a long-term period?

**FIGURE 2:**
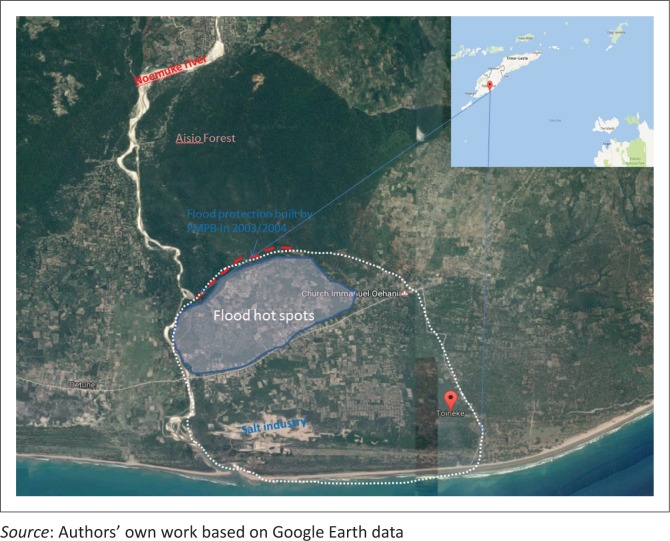
Toineke village in geographical context.

Rather than exhibiting a sophisticated analysis, the article combines storytelling as well as reflections informed by 20 years of CBDRR engagements with the communities in Toineke, a small delta village situated in Kualin sub-district in South Central Timor (TTS), eastern Indonesia (Figure 2). The story of the evolution of risk management goes back six decades in the past.

## Methods

This research uses longitudinal observations and combines qualitative and quantitative methods. The qualitative approaches are based on longitudinal observation during 1999–2017. Field observations by the authors occurred during several flood and drought events between 1999 and 2010. Data collections have been a mixture of project assessments, monitoring and evaluation as well as unpublished reports (Table 1). The *Perhimpunan Masyarakat Penanganan Bencana* (PMPB, Community Association for Disaster Management), (formerly known as Forum Kesiapan dan Penanganan Bencana [FKPB] or Disaster Preparedness and Response Forum) in West Timor conducted two participatory assessments in 2001 (using Participatory Rural Assessment methods) and 2002 (focused group discussions and community meetings), followed by several updates in 2004, 2007 and 2010. Participant observations have been conducted several times during 2001–2010.

**TABLE 1:** Mixed methods.

Methods	Approach/source	Remarks	Timeline
Qualitative	Participatory action and learning	Risk mapping, food choice map, welfare ranking, coping mechanism, priorities and ranking	2001, 2002
	Oral history	Elders as informants	100 year scale
	Media report	-	2000–2017
	Social media	Facebook	2012–2016
	Discourse analysis	Village planning	2011–2015
	Secondary data	Situational reports	1999–2010
Quantitative	Household survey	Food and livelihoods monitoring survey	2007
	Statistical data	Secondary data	Village data 2000–2010; BPS data 2014–2016

BPS, Badan Pusat Statistiks.

This research is also informed by many unpublished reports that have been drafted by both the PIKUL Foundation and PMPB during 2000–2010. The authors have been visiting this village numerous times in the last 18 years to understand social and environmental changes and challenges in the Toineke village in West Timor, Indonesia, in the last 40 years. Numerous documentations were made during this period (e.g. Ahalamani & Pelokila 2010; Boli et al. 2007; Lassa 2005; Leonis et al. 2004; Nakmofa & Lassa 2009).

Some of the first visits were part of a humanitarian emergency responses to El-Niño-induced droughts and floods in 1999 and flash flood emergencies in 2000 (led by 2nd and 4th authors). Forum Kesiapan dan Penanganan Bencana conducted a community-based flood management programme during 2000–2001 and later 2002–2004 including flood emergency assessments in 2003–2004 and capacity building by an NGO, namely PMPB, via its food and livelihoods monitoring system (FLMS) that had been implemented during 2004–2008. FLMS involved household surveys and regular monitoring in the village. The authors recently used social media (Facebook) to monitor several mentions of Toineke during several flood events that occurred during 2012–2017. The interesting part of this method is that it can monitor several flood events in Toineke with recorded videos and photos posted online. Media archives (clippings) from 2012 to 2016 have been used to validate records from social media for both flood and drought events in Toineke.

## Context

### Brief history of *Perhimpunan Masyarakat Penanganan Bencana* Kupang

Responding to the strong El-Niño event during 1997–1998, local civil society formed a Food Insecurity Information Centre (PIRP, *Pusat Informasi Rawan Pangan*). It was established to help local communities, local governments, NGOs and United Nations agencies with alternative information on potential hunger and food insecurity including potential widespread of malnutrition in East Nusa Tenggara (NTT) province, Indonesia. The severe droughts triggered by one of the strongest El-Niño events in modern Indonesia affected the quality of life of the people in West Timor. The event had been exacerbated by the onset of Asian economic crisis during 1997–1998 that led to Indonesian political crisis that eventually led to the fall of Suharto regime. The cascading effect of political-economic crisis led to the independence of East Timor from Indonesia. The processes that surrounded the independence of East Timor had been pre-empted by a major humanitarian crisis in East Timorese which led to the influx of 300 000 refugees to West Timor, Indonesia.

The civil society forum in West Timor later changed the mandates of PIRP by expanding its focus from famine and food insecurity towards all types of hazards. Such transformation led to the changing of names from PIRP to FKPB to PMPB (Hereinafter, this article will refer to PMPB).^1^

### Brief background of Toineke village

Etymologically, Toineke is derived from *toi* [gate] and *neke* ([kapok tree] Java cotton or *Ceiba pentandra*). Kapok tree has been used to gain cash not only in bad years (during long severe drought) but also in good time when the harvest of food crops was good. The villagers have been producing kapok cotton and sold it to Chinese traders at least in the last 80–100 years. Despite its physical proximity to the southern sea of West Timor (Figure 2), situated at 1 m – 15 m above mean sea level (MSL), the communities’ livelihoods orientation mainly centred on dryland agriculture. Fisheries is the least developed sector because most West Timorese in the village and elsewhere do not have the skills and culture for offshore fishing.

Under the agro-ecosystem classification, Toineke village is classified as having an alluvial system where its soil is mostly sandy clay. Situated in the dryland of the south coast of South Central Timor, the village is famously known as a disaster-prone area. Annually, the Toineke and its surrounding areas receive less than 500 mm rainfall spanning only 4–5 months, mainly during December–May. The Aisio Forest has provided multiple ecosystem services ranging from flood protection, food security during drought and livelihood diversification. Since the occupation of the Toineke landscape up to the early 1980s, the Aisio Forest has been the natural flood protection for the early settlers of Toineke. Local communities have been harvesting *candlenut, tamarind, kapok and* other ‘forest’ commodities from Aisio. In the last 30 years or more, Toineke has been a ‘business hub’ where weekly traditional markets are held every Saturday. In bad times (e.g. long severe droughts), this market has been one of the institutions (in the logic of institutional studies) or mechanism where the locals exchange their crops as well as non-timber forest products (NTFPs) (from their gardens as well as from the nearby forests) in return for cash, rice and other household goods.

Recent annual trends shows that the village is growing at 1.5%. The village population has increased from 2232 people in 2002, 2688 people in 2009 and 2696 people in 2015. The total land area, excluding forests, is 27.5 km^2^ as informed by the local communities and village government during PMPB assessment. As of 2007, 320.0 ha land has been used for gardening and housing; 513.5 ha was used for agricultural plots, while the rest are uncultivated land and forest. In recent data from *Kualin in Figures* 2015 and 2016, the land size included the forest area which brings a total of 39.3 km^2^ for the whole village.

## Results and discussion

### Community perception of risk and hazards

Disaster specialists are often interested only in natural hazards such as floods and droughts. However, the communities have been concerned with some of the more regular problems including the everyday problems (Bogardi et al. 2009) such as lack of financial capital to facilitate access to better cropping technology, lack of skills and human resources, price volatility and lack of market access and lack of control to wages.

Using cardinal ranking logic, Table 2 suggests that flood and flood inundations are the most prioritised events. Droughts comes after the floods followed by pests and diseases. Apart from these three hazards, the local communities have been worried about their need to get access to financial capital (e.g. credit access and technological access), skills, price volatility, market access and wages. The community has been facing immediate risk of price volatility because of their close proximity to seasonal trade every Saturday. Some farmers went to the nearby cities to sell their labour and often felt that wages are not properly created and just.

**TABLE 2:** Hazard and problem ranking.

Number	Type of hazards or problem	Spread or distribution	Magnitude	Frequency	Sum	Rank (cardinal system)
1	Pest and diseases	3	2	3	8	II
2	Drought	3	3	2	8	II
3	Flood and flood inundation	3	3	3	9	I
4	Lack of capital	1	3	3	7	III
5	Lack of skills	1	1	3	5	IV
6	Price volatility	1	3	3	7	III
7	Market access	1	1	3	5	IV
8	Wages	1	3	3	7	III

*Source*: Risk and problem ranking, (PMPB, 2002, *PRA Toineke village*, Unpublished report, PMPB, Kupang)

For spread or distribution: 3 = all village affected; 2 = partial and 1 = only a fraction of the area get affected.

For magnitude: 3 = the event is very big, 2 = modest and 1 = small.

For frequency: 3 = occurs every year; 2 = 3–4 year event; 1 = the event seldom occurred.

Cardinal ranking means the participants summed.

The communities identified each type of problem defined in Table 2 while indicating actions being taken before and after 2000 (Table 3). Table 3 suggests that the involvement of NGOs and other external actors has been facilitating empowerment to some degree where the communities transformed themselves from non-intervention state towards building flood walls and planting trees.

**TABLE 3:** Hazard and problem ranking.

Number	Identified problems	Pre-2000	Post-2000	Remarks	Source of information
1	Flood inundations and flood risk	Non-intervention – but late 1990s an NGO tried to mobilise the local communities to build drainage but failed	Build flood wall (embankment) to channel back to the river (2000–2001; 2003–2004) Plant trees along the flood wall (2006)	Most flood wall co-constructed by PMPB and the villagers have been damaged and less functioned Trees mortality was not properly counted/addressed systematically	PMPB (2002); PMPB (2007)
	Food shortage associated with floods	Harvest palm; tamarind seeds and wild tubers (wild sweet potato)	On top of the past adaptation, the villagers expect to get subsidised rice	The good news is that subsidised rice seen as bonus	PMPB (2002); PMPB (2007)
2	Drought	Pray for rainfall	Pray for rainfall Sales of NTFP products villagers expect to get subsidised rice Local government provide small dams	Some communities have been able to utilise small dams. Dynamics of local arrangement of the use of these dams are still unknown	PMPB (2002); PMPB (2007); PMPB (2010); Fieldwork in 2008 (Lassa)
3	Pest attack and crop disease	Traditional ceremony	Pesticide use	No longer conduct traditional ceremony because of the passing away of elders	PMPB (2002); PMPB (2007)
4	Capital, marketing and price risk	Lower price during harvest; price volatility; lack of access to financial capital	In situ marketing of crops and NTFPs products during Saturday market; higher penetration of financial and credits services from outside	Better transportation Good transportation Volatility in production persist	PMPB (2002); PMPB (2007)
5	Labour and technology	Work manually	Still work manually	Lack of capital block access to technology	PMPB (2002); PMPB (2007)

*Source*: PMPB, 2002, *PRA Toineke village*, Unpublished report, PMPB, Kupang; PMPB, 2007, *PRA Toineke village*, Unpublished report, PMPB, Kupang

Note: Please see the full reference list of the article, Lassa, J.A., Boli, Y., Nakmofa, Y., Fanggidae, S., Ofong, A. & Leonis, H., 2018, ‘Twenty years of community-based disaster risk reduction experience from a dryland village in Indonesia’, *Jàmbá: Journal of Disaster Risk Studies* 10(1), a502. https://doi.org/10.4102/jamba.v10i1.502, for more information.

NGOs, non-governmental organisations; NTFPs, non-timber forest products; PMPB, *Perhimpunan Masyarakat Penanganan Bencana*.

### Severe drought and El-Niño

Most of the villages in the southern coast of West Timor have a dry climates. Small changes in the climate in Timor Sea will affect the villagers. In this context, El-Niño Southern Oscillation (ENSO) often triggered severe droughts. This has been observed in Toineke as the local communities remembered some of the past events during 1965, 1997, 1998, 2000, 2002, 2005 and 2007. We found that local governments have improved their disaster response capabilities in recent years. For example, in the recent ENSO-driven droughts in 2010 and 2015, the droughts did not lead to emergencies because there was adequate response from the local governments (see Table 4).

**TABLE 4:** Drought-related hunger and famine history in Toineke.

Number	Famine and hunger events	Trigger	Impact	Coping
1	1938–1940	Long severe drought	Harvest failure; crop loss	Temporary migrate to original village to farm; exchange livestock with food
2	1964–1965	Long severe drought	Harvest failure; widespread crop loss	Harvest palm trees; wild tubers; exchange livestock with food
3	1970–1971	Moderate drought	Harvest failure; widespread loss	Harvest palm trees; wild tubers; exchange livestock with food; selling NTFP products to traders
4	1972	Pest attack, locust attack and moderate drought	Harvest failure; widespread crop loss	Harvest palm trees; wild tubers; exchange livestock with food; selling NTFP products to traders
5	1997/1998	Strong El-Niño	Harvest failure; widespread crop loss	Food aid in combination with harvest palm trees; wild tubers; exchange livestock with food; selling NTFP products to traders
6	2007	Drought	Moderate crop loss	Replanting; harvest palm trees; wild tubers; exchange livestock with food; selling NTFP products to traders
7	2010	Moderate drought	Moderate crop loss	Access to Raskin (food subsidy)
8	2015	Strong El-Niño	Severe drought; harvest failure; crop loss	Access to Raskin (food subsidy) – despite late response

*Source*: PMPB, 2002, *PRA Toineke village*, Unpublished report, PMPB, Kupang; PMPB, 2007, *PRA Toineke village*, Unpublished report, PMPB, Kupang; and media reports, for example, Pos Kupang.

NTFP, non-timber forest product.

Very often, droughts caused reduction of consumption quality and quantity leading to food insecurity and malnutrition (Table 4). The risk can be compensated by trading NTFPs. The villagers have gained access to NTFPs such as *kapok* cotton and tamarind seeds and tamarind meat. Interestingly, based on village reports as of 23 October 2003, there have been recorded sales of 250 metric ton tamarind from Toineke (and equivalent of Rp. 250 million or $30 000). The sales of kapok cotton hit 40 metric ton (Rp. 40 million or $5000.00). It means during September–October 2003, there has been recorded sales amounting to $35 000.00 from the village. Such capacity is often not recognised from formal reports of local governments as well as NGOs.

Combining NTFPs, cash economy and subsistent agriculture have been the key strategy to build resilient livelihoods in Toineke. The villagers often harvest palm trees to eat like sago or *putak* that often cooked together with beans and tubers not only during bad years but also during ‘normal’ years.

### Flood history

Floods have been disruptive to their livelihood assets such as livestock and food crops. Toineke flood news has been constantly hitting local and national media, especially during severe floods and droughts in the last 20 years. Harvest losses and food insecurity have been the most common phenomena, especially in the northern side of the village. The situation has been exacerbated by the lack of proper road drainage. During flood season, the northern part receives excessive run-off that cannot be discharged to the sea as the road prevents the water from flowing to the coasts. Verbal history narrated by the communities suggested that the first damaging floods started in 1982 when the road construction finished. Toineke’s soil is alluvial type; so in order to have longer lifespan of services, the road must be constructed at certain height. The problem is that most rural roads and highways in Indonesia do not have drainage channels. As a result, surface water run-off often inundated both agricultural fields and community housing during flood seasons.

**FIGURE 3:**
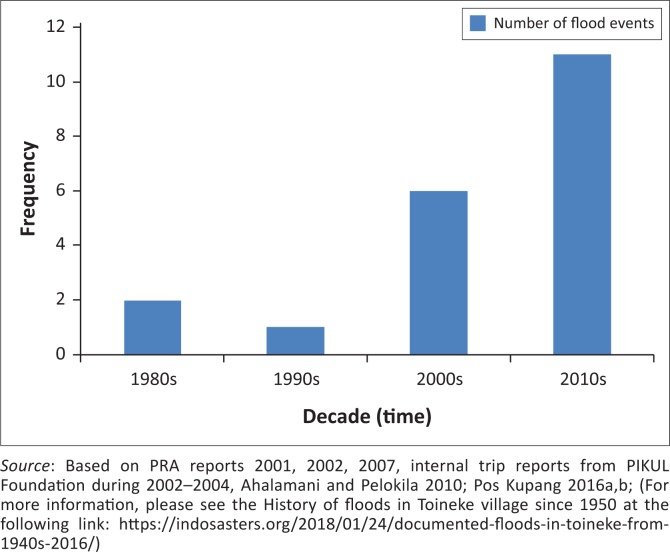
Flood events and trends between the 1980s–2010s (flood frequency).

The historical events from 1982 were coincidental with the road – it was elevated and paved but no drainage channel was constructed (Lassa 2018). Most elders narrated that this ignorance of the need of drainage continues to happen till today. Figure 3 shows the trend of flood events in the last four decades since early 1980s. It shows that the village has continued to experience more flood events as it is in fact getting worse from time to time. The floods in 1991 had triggered a widespread contamination of water because of lack of protection of the wells. Most of the wells are not protected by concrete cover. The 2000 floods have been the worst in history where widespread diarrhoea caused the death of 22 children aged under 5.

### Lessons from community-based flood management project

In 1999, FKPB visited the village as ‘the lord of the poor’ by responding heroically to lessen the severity of the drought impact in 1998 amplified by one of the strongest El-Niño in decades. The response was justified by sudden declines of food security and the quality of life of the villagers. Therefore, instead of taking the offer from one philanthropist from Japan to buy a vehicle for daily operational, the activists at FKPB and/or MPB healthfully used the earmarked fund for an office car to buy chickens for the villagers to improve their livelihoods. The final distribution took place in December 1999, followed by post-distribution monitoring in early 2000. On 16 May 2000, a flash flood hit the village because of continuous rainfall starting late April 2000 up to the first week of May 2000. Most of the deltas in the southern coasts of West Timor had been badly damaged by heavy storms and continuous rainfall. Such rainfall behaviour could have been triggered by the Rosita Cyclone that formed less than 100 km off the Timor Sea. All the chickens distributed by FKPB were swept away by the floods. A cholera outbreak post-flooding caused the death of 22 children aged under 5 years of age because of a lack of protection from the water sources during the flood inundation.

*Perhimpunan Masyarakat Penanganan Bencana* (PMPB) allocated some of the donated money for the flood mitigation project to help the people organise themselves by clearing up access to the embankment. PMPB worked together with the communities to build about a 6 km embankment along the river bank to partly prevent future flood inflow to the village. Because of lack of support from the local communities, PMPB later hired a small size tractor to dig and make the embankment. To get the people mobilised to support the construction of the embankment has proved to be difficult. PMPB was frustrated by the delays of response from local communities. PMPB tended to think that the Toineke community was passive. PMPB decided not to leave the village because at that time most NGOs pulled out from the areas after their El-Niño project ended (PIKUL Foundation 2007).

Unfortunately, the embankment was short-lived as the quality of the construction was compromised. The NGO activists maintained the view that an embankment that is not formed as a straight line is a problem. The embankment was bended because one villager rejected the idea to build the embankment via his land. The embankment collapsed by the flood that occurred in the 2001. Flood water caused scouring and eroding of the base of the embankment. Extremely high sediment transports quickly to pill up and compromised the function of the embankment.

The approach from ‘food for work project’ from international operations during 1998 El-Niño crisis had been partly blamed by the elders as the root cause behind the non-participation of the local communities in joining efforts to build a proper drainage using self-help approach. The risks alone did not unite the people to solve their shared problems. Lack of food and cash-based incentives delay collective actions. The NGOs have been struggling between the idealism to cultivate genuine participation from the local communities to repair the embankment; however, the non-response from the local communities prevailed.

*Perhimpunan Masyarakat Penanganan Bencana* later continues this ‘Community Based Flood Mitigation (CBFM)’ project funded by PIKUL Foundation during 2002–2004. Floods have damaged the embankment built in 2003–2004. Non-governmental organisations have been trying to fix the problem on the spots, and this has been not effective. On the spot, we also observe that the local government has been trying to solve the problem by constructing artificial riverbank since the late 1990s. However, the flood problem remains as annual problem till date. Nahak et al. (2008) argued that floods can be solved in a more systematic way such as by creating artificial basins and/or retarding basin that provides risk reduction services by temporarily holding water in land and slowing down destructive flow of water directly to the village.

### Adaptation to floods: A positive deviation story

According to the data from Toineke (2011), the village has at least 180 wells not including 8 boreholes. Cholera and diarrhoea outbreaks in 2000 were particularly associated with unprotected wells. Therefore, the standard response from PMPB as a disaster response NGO was mainly to limit the spreading of the risk by protecting the wells. The problem was that most of the capped wells constructed in early 2000s were no longer effective.

We found that some of the villagers have been trying to protect their wells by elevating the cap wells higher than the elevation of inundated flood waters. Some of these observations were made during and after a flood on 13 April 2003 where we found that about 150 families evacuated themselves to a safe haven, a higher zone in Aisio Forest. The authors (Lassa and Leonis) observed a day later that most families did evacuate their livestock such as chickens, pigs and goats as well as cows with some kitchen utensils as well as strategic assets such as jewellery. The main reason was to save their livestock as strategic assets.

As Toineke’s climate and weather has been influenced by the behaviour of Timor Sea which often hosted several cyclonic events, the village often gets its ‘stormy season’. The locals call it the ‘west monsoon season’, a stormy period where the Manu family said that it is often the time to put their goats and pigs and chickens in the cages as their household anticipatory action. However, their cattle are kept on higher grounds.

We asked one of the villagers who decided not to evacuate in 2004 and his response was (Nakmofa & Lassa 2009):

‘Why I have to evacuate? My son, I have observed maximum level of flood that can enter my field over the course of years. Look at the water level now; it only reaches 40 cm while the topping of my wells is far higher than the water level. Come and see my pig and chicken stall, I have made the stall level far higher than the water level I have observed to enter almost 40 years I live here. I have also increased the level of the flood house, so the water could not enter. So, on what I account I should evacuate my family?’ (p. 144)

### Coping mechanism – Combination of food diversification and reduction of food intakes

Corn is the main food for the people. Cash from the sales of NTFPs and livestock is often used to procure corn and rice. Despite the diverse source of food, our observation suggests that there have been cases where households need to rely on external supports such as rice distribution from local governments and/or NGOs as they run out of food sources.

During several meetings and consultations, the community members have consistently mentioned about the practice of reducing food intakes – for example, from three meals to two meals in a day as part of coping mechanism (Lassa 2009). Some families noted that the decline of palm trees has pushed the community members to sell their small livestock in order to buy foods. During a field visit in 2009 and 2010, it was observed that some households tried to reduce their food intake from December (in 2009) till February (in 2010) when new harvest season started. Reduction of the frequency of food intake can also mean several layers: firstly, reduction from 3 to 2 meals; secondly, temporary elimination of snack and coffee in the morning; thirdly, sales of small livestock for rice; and last, rice is cooked as porridge for kids.

### Government intervention – Safety nets and village funds

Government’s subsidising rice has been instrumental in avoiding a food crisis in the village. Such approaches are not enough as welfare losses still occur due to floods and droughts. As of 2015, 392 families are the recipient of the safety net, namely rice for the poor. The total village fiscal in 2015 was Rp. 642 million (about Rp. 900 000 per family or Rp. 240k per person per year) (BPS 2015). The detailed allocation of these funds is not entirely understood. The challenge is how to use the recently allocated village funds in a way that improves the quality of life as well as building resilience to floods and droughts.

### Floods and social media observation during 2012–2017

Despite being voluntary to drought risk, there has been a reduction in their vulnerability as both internal and external interventions have been made to lessen the risks and impacts of droughts. There is criticism from politician and NGOs concerning the increasing fragility of the village towards flood and drought shocks. Recent observations in 2012, 2013 and 2016 suggest the fact that local government has been more responsive. Both media and social media helped spread the news, and the local disaster management authority (BPBD) as well as the head of the district could visit the village within 48 h in the case of floods.

## Conclusion

As early adopters of CBDRR, we observed that the approach has been introduced by non-governmental organisations (NGOs), including our organisations in Indonesia since late 1990s. Such a big push by NGOs was aimed at recognising the roles of local communities and local capacities. In addition, there has been an increasing trend of disaster risk governance practice in Indonesia in the last 50 years (Lassa 2013) where both local and national governments have been able to promote and pilot disaster resilience building at village level (BNPB 2015). The outcomes of CBDRR vary from places to places.

As answer to the question ‘how can a CBDRR project contribute to long-term social change?’, we notice and celebrate incremental changes in Toineke. We witnessed some good practices from particular households and community members. Some local community members have been able to cope with the floods and droughts in the last 20 years. Some forms of adaptations such as wells’ protection are not entirely spontaneous. We also noticed that PMPB’s intervention in early 2000 to concretise the wells as well as to construct flood embankments was not sustained because of the increase in both frequencies and scales of impact. In addition, most of the changes are the outcomes of complex interactions between all the actors from within and outside the local communities.

Understanding the emergence of the outliers such as the adaptation practices in Section ‘Adaptation to floods: A positive deviation story’ required long-term engagement with local communities to allow consistent and thorough observations. The challenge is how to scale up some of these practices to wider scale. Our recent observation suggests an incremental change in flood risk management and adaptation. Social replication of certain adaptations such as wells’ protection and improvement as well as elevating houses’ to prevent flooding have become a real-world practice (see the visual documentation of a recent visit by the first author to the village) (Lassa 2018b). Furthermore, access to diversified resources such as NTFPs has been one of the endogenous elements in community’s resilience to droughts and floods. Some of the challenges for the future are that with the increasing population projected to increase by 2050, how the communities rearrange their access to land use, access to forest, and competition over free flood zones.

In general, facilitated via the participatory processes, the elders generally could identify the root causes of risks and vulnerabilities that evolve in the village over long period of time. In some ways, NGOs and local communities believe that the declining of Aisio Forest Ecosystem Services has been like a leaking boat where most of the efforts to fix the holes are about delaying the end of the history. However, this alarmist view might not fit with the real progress and achievements. Overall, there is a declining trend in flood disaster and drought mortality in Toineke. Recent global trends suggest that while there is a consistent decline in global disaster mortality, however, both mortality risk and economic loss from localised disaster events are increasing (UNISDR 2015:4). These have been in particular associated with the human dimension of vulnerabilities ranging from inequality, environmental degradation and weak governance. The absence of rural planning and/or badly planned rural shelter (Dasgupta et al. 2014) has been contributing to rural risks.

In Toineke, we argue that rural planning needs to be moving beyond agriculture to include shelter, education, nutrition and social policy. This planning must capture the complexity of people’s life and livelihoods. Unfortunately, we also observe that during the recent floods in 2012, 2013 and 2016, the local schools have been affected, as one high school teachers and students posted that their books and tables have been flooded. The current response system has been mainly focused on a small fraction of local livelihood systems. It needs to address the issue of education in times of flood emergencies. Furthermore, increasing vulnerability to floods and droughts has its implication on nutritional issues. Most often, nutrition disappears from communities’ risk ranking and problem identification. The challenge is how to train future community development workers and emergency responders as well as agriculture officials that can be mindful of all the complexity of building community resilience.

Toineke’s village planning document noted the fact that 12 km out of its 19 km unpaved road was broken as a result of 2012 floods, and about 1 km out of its 7 km asphalt road was damaged, while its 4 km paved road was all damaged (Toineke 2011). Unfortunately, the document made no explicit references to floods at all. This suggests that local communities did not fully adopt the risk assessment results to inform their recent planning documents. However, this is not surprising because most of these documents have been outsourced and completed by external consultants who might be either blind to disaster risks issues or simply using a general national template that is skewed towards big projects such as roads that had recently been funded by both local government as well as international donor projects.

While trying to change the community, we ended up changing our perception about social change. We found that social change takes longer than expected by NGOs’ and donor’s project log-frame (or theory of change) documents that often unrealistically demand for change in a very short time. Non-governmental organisation workers (as most of the authors used to be) are often pushed for prompt public action while building and nurturing the condition for collective action from the communities. Nurturing community and collective action is not a simple endeavour. Our 15 years of observation have improved our understanding of reality of risk as well as the communities. Spending decades not guarantee continuity of collective action and trust; but without intimately working with communities, chances for social change and future collective action might be very small.

We finally conclude that CBDRR concepts and procedures could provide general guidelines for grassroots’ actions. However, not all the prescribed elements (Figure 1) can be accommodated and funded because of a lack of resources. Community-based approach required a more relaxed and informal approaches, and the progress can manifest in chains of *ad hoc* success and failures, involving trials and errors (Macherera & Chimbari 2016). The final stages such as evaluation and monitoring, including exit strategy could not always be carried out. Apart from the issues of local NGOs’ financial capacities, their close proximity to local communities made us realise that entries and exits to and from at-risk communities are often blurred. Such framing (of entry and exit) might be fit for international NGOs and donors but a vague concept for local governments as well as local NGOs.

## Footnotes

1 Three of the authors used to be key NGOs’ activists from West Timor, Indonesia, who have been funded by Oxfam to be trained in community-based disaster risk reduction by Asian Disaster Preparedness Centre (ADPC) in both Bangkok and Manila during 1998–2002. However, as this article shows, some local NGOs ended up continuing engaged with an at-risk community for 12 years in a row followed by further engagements via assessments, research and later on social media.
